# De Novo Noninversion Variants Implicated in Sporadic Hemophilia A: A Variant Origin and Timing Study

**DOI:** 10.3390/ijms25031763

**Published:** 2024-02-01

**Authors:** Ming Chen, Ming-Ching Shen, Shun-Ping Chang, Gwo-Chin Ma, Dong-Jay Lee, Adeline Yan

**Affiliations:** 1 Department of Genomic Medicine, Changhua Christian Hospital, Changhua 500, Taiwan; 104060@cch.org.tw (M.C.); 70914@cch.org.tw (S.-P.C.); 128729@cch.org.tw (G.-C.M.); 118862@cch.org.tw (D.-J.L.); 181435@cch.org.tw (A.Y.); 2 Department of Obstetrics and Gynecology, Changhua Christian Hospital, Changhua 500, Taiwan; 3 Department of Medical Genetics National Taiwan University Hospital, Taipei 100, Taiwan; 4 Department of Obstetrics and Gynecology, National Taiwan University Hospital, Taipei 100, Taiwan; 5 Department of Laboratory Medicine, National Taiwan University Hospital, Taipei 100, Taiwan; 6 Department of Internal Medicine, National Taiwan University Hospital, Taipei 100, Taiwan; 7 Hemophilia Treatment and Thrombosis Center, Department of Internal Medicine, Changhua Christian Hospital, Changhua 500, Taiwan

**Keywords:** de novo variant, noninversion variant, sporadic hemophilia A, hemophilia A, mosaic variant, germline mosaicism

## Abstract

Sporadic hemophilia A (HA) enables the persistence of HA in the population. *F8* gene inversion originates mainly in male germ cells during meiosis. To date, no studies have shown the origin and timing of HA sporadic noninversion variants (NIVs); herein, we assume that HA-sporadic NIVs are generated as a de novo variant. Of the 125 registered families with HA, 22 were eligible for inclusion. We conducted a linkage analysis using *F8* gene markers and amplification refractory mutation system–quantitative polymerase chain reaction to confirm the origin of the sporadic NIVs (~0% mutant cells) or the presence of a mosaic variant, which requires further confirmation of the origin in the parent. Nine mothers, four maternal grandmothers, and six maternal grandfathers were confirmed to be the origin of sporadic NIVs, which most likely occurred in the zygote within the first few cell divisions and in single sperm cells, respectively. Three mothers had mosaic variants, which most likely occurred early in postzygotic embryogenesis. All maternal grandparents were free from sporadic NIV. In conclusion, *F8* NIVs in sporadic HA were found to be caused primarily by de novo variants. Our studies are essential for understanding the genetic pathogenesis of HA and improving current genetic counseling.

## 1. Introduction

Hemophilia A (HA) is an X-linked congenital bleeding disorder in humans caused by the deficiency or dysfunction of factor VIII (FVIII) [1]. FVIII functions as a cofactor for activated factor IX (FIX) in the activation of factor X (FX) [2,3]. The gene coding for *F8* is located on chromosome Xq28, spans 186 kb, consists of 26 exons, and encodes a protein comprising 2332 amino acids [4]. HA gene variants can be categorized as either inversion variants, consisting of intron 22 and intron 1 inversions in the *F8* gene, or noninversion variants (NIVs), such as point variants, deletions, insertions, or duplications [5,6,7]. Based on plasma FVIII activity, HA is clinically classified as either mild (>5–40 IU/dL), moderate (1–5 IU/dL), or severe (<1 IU/dL) [1].

For thousands of years, the life expectancy of patients with hemophilia (PWH), especially those with severe cases, has been very short due to the lack of appropriate medical care [8,9]. Thus, PWH might gradually decrease in number in the population; however, newly diagnosed hemophilia cases develop when genetic variants causing hemophilia occur spontaneously [8,10], where the latter occurs even in the present era of medical care. We were interested in understanding how these newly diagnosed sporadic HA genetic variants occurred and sought to identify their origin. Furthermore, we aimed to determine when the new genetic variant might occur during embryogenesis for a better understanding of additional genetic pathogenesis. Sporadic hemophilia is the first affected PWH observed in a family where no relative or cousin has hemophilia or has been proven to be a hemophilia carrier [8]. *F8* gene inversion has been studied, with evidence showing that *F8* gene inversions originate mainly from male germ cells during meiosis [11,12]. Therefore, in this study, we studied only HA families bearing sporadic NIVs. We assumed that the Hemophilia A sporadic noninversion variant is caused by a de novo variant (DNV). DNV is defined as a variant observed in a child but not in either of the parents [13,14]; accordingly, the family member designated as the DNV origin must be free of coagulation alteration and genetic variants associated with DNV. Furthermore, individuals with mosaic variants undetected owing to the use of less sensitive conventional methods are not considered DNV origins [14]. Sporadic hemophilia is a clinical term that refers to hemophilia with a negative family history, and de novo hemophilia denotes the most likely occurrence of a new hemophilia gene variant without familial transmission. Previously, there were several studies reporting about DNV association with sporadic HA genetic variants [8,15,16,17], but the definition of DNV was not well defined and the association of sporadic HA genetic variants with DNV might not be well studied. To the best of our knowledge, to date, no studies have reported the real origin and timing of hemophilia A sporadic noninversion variant.

This study aimed to (a) confirm that hemophilia A sporadic noninversion variant is caused by DNV; (b) understand the genetic pathogenesis, which is crucial for improving current genetic counseling; and (c) provide accurate genetic counseling and evidence concerning who in the sporadic HA family is the origin of a sporadic genetic variant and who is the occurrence of a sporadic genetic variant. This study will show that an X-chromosome from a family member who is designated as the origin of a sporadic genetic variant and originally bears a wild-type HA gene transmitting to his/her offspring becomes mutated and bears the pathogenetic variant of the HA gene during the embryogenesis of his/her offspring, who will be designated as the occurrence of the sporadic genetic variant. If the HA genetic variant identified in an offspring is inherited, similar genetic variants should also be identified in his/her parents and older generations. We verified that HA-sporadic NIVs were primarily caused by DNVs. This finding contributes to creating a better understanding of the genetic pathogenesis of HA.

## 2. Results

### 2.1. Characteristics of Patients with HA

Based on the criteria of inclusion that required families to have sporadic HA and NIVs as well as difficulty in acquiring samples from family members, only 22 of the 125 registered families were suitable for inclusion in this study. Twenty families from our hemophilia centers and two referred families were recruited for the study. As shown in Table 1 and Table 2, 18 patients were diagnosed with severe hemophilia, 3 with moderate hemophilia, and 1 with mild hemophilia. Patient ages were 0.1–57 years, with a mean and median of 17.3 and 10.5 years, respectively. The genetic variants that were observed included twelve point variants, of which eight were missense variants and four were nonsense variants, two splice-site variants, four duplications, and four deletions.

### 2.2. Linkage Analysis and Genetic Testing for Identification of the Possible Origin of Sporadic HA Noninversion Variant and Amplification Refractory Mutation System–Quantitative Polymerase Chain Reaction for Confirmation of Sporadic NIV Origin

#### 2.2.1. Studies in Families 1 to 19 Who Have Sporadic HA NIVs without Mosaic Variants

**Figure 1:**
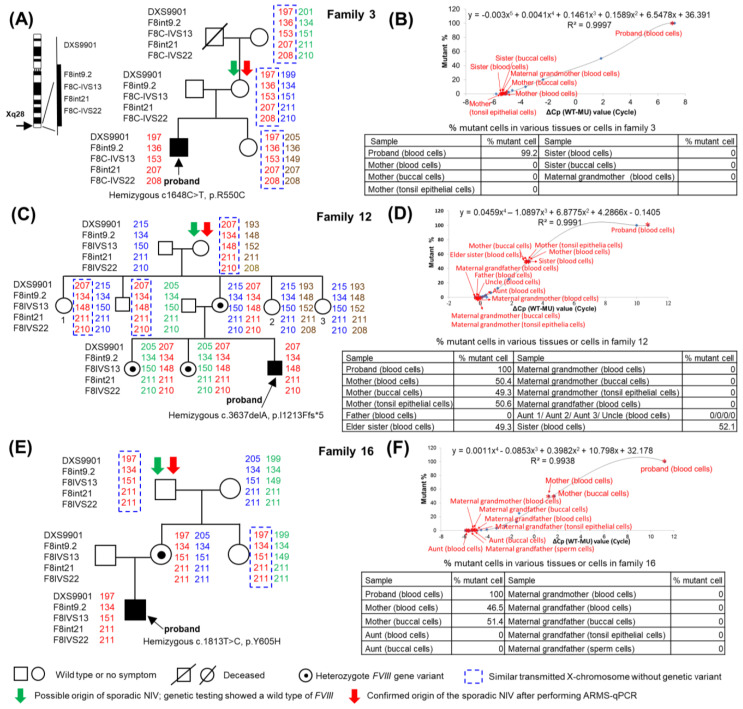
Linkage analysis using intra- and extragenic markers for the *F8* gene, genetic testing, and amplification refractory mutation system–quantitative polymerase chain reaction (ARMS-qPCR) to confirm the origin of the sporadic noninversion variants (NIV) in families 3, 12, and 16. (**A**,**C**,**E**) Linkage analysis using *F8* gene markers in family 3, severe hemophilia A; family 12, severe hemophilia A; and family 16, severe hemophilia A, respectively. The *F8* gene located on the X chromosome (Xq28) is shown on the left side of (**A**), along with the four intragenic and one extragenic markers (F8int9.2, F8IVS13, F8int21, F8IVS22, and DXS9901) selected for linkage analysis. (**B**,**D**,**F**) Determination of the percentage of mutant cells using ARMS-qPCR in various tissue cells obtained from family members to confirm the origin of the sporadic NIVs (~0% mutant cells) or the presence of somatic and/or germline mosaic variants (0–50% mutant cells) in families 3, 12, and 16, respectively. ΔCp (WT − MU) represents the differences between the qPCR cycle crossing points (Cp) of the wild-type (WT) and mutant (MU) alleles of different synthetic dilutions (X−axis). Blue points (◆) indicate various synthetic dilutions prepared via a two-fold serial dilution of MU DNA using WT DNA, as described in the text. The standard curve shown in the upper part of each figure was generated by plotting the ΔCp value (X−axis) against known mutant percentages of various synthetic dilutions (Y−axis). Thereafter, the equations for X and Y were derived. When ΔCp (X) of the test sample after ARMS-qPCR was known (shown in red asterisks), Y (% mutant cells) was calculated from each equation as shown in each table.

Linkage analysis and amplification refractory mutation system–quantitative polymerase chain reaction (ARMS-qPCR) were performed on tissue samples from multiple family members in each family. For example, as shown in Figure 1, mothers and maternal grandparents whose *F8* STRs were of a similar size to that of the proband (shown in red) were considered to have the same transmitted X chromosomes. Any family member who had a wild-type (WT) *F8* derived from the mother or maternal grandparents would be designated as the possible origin of the sporadic NIV (shown in the green arrow). For example, the mother in Figure 1A, the maternal grandmother in Figure 1C, and the maternal grandfather in Figure 1E were designated as the possible origins of sporadic NIV.

The results of the ARMS-qPCR assay, as shown in the small tables in Figure 1B,D,F, revealed that the percentage of mutant cells was approximately 100% in the blood cells of the proband of these three families, as expected. However, 0% mutant cells in various tissue cells were detected in the mother of family 3, the maternal grandmother of family 12, the maternal grandfather of family 16, and the other family members, as well as in the sperm cells of the maternal grandfather of family 16. In contrast, approximately 50% of mutant cells were found in various tissue cells of both mothers from families 12 and 16, as well as in both sisters of family 12, who were heterozygous for the *F8* mutants. These results suggest that the confirmed origin (shown in red arrow) of the sporadic NIV was the mother, maternal grandmother, and maternal grandfather of families 3, 12, and 16, respectively. However, no germ cells were available for study from the mother of family 3 or the maternal grandmother of family 12.

Table 1 summarizes the family members designated as the possible origin of sporadic NIVs as determined via linkage analysis and genetic testing in each family (families 1–19). The identity of the origin was further confirmed by ARMS-qPCR, which showed 0% mutant cells in various tissue cells.

#### 2.2.2. Studies in Families 20–22 Who Had Mosaic Variants

**Figure 2:**
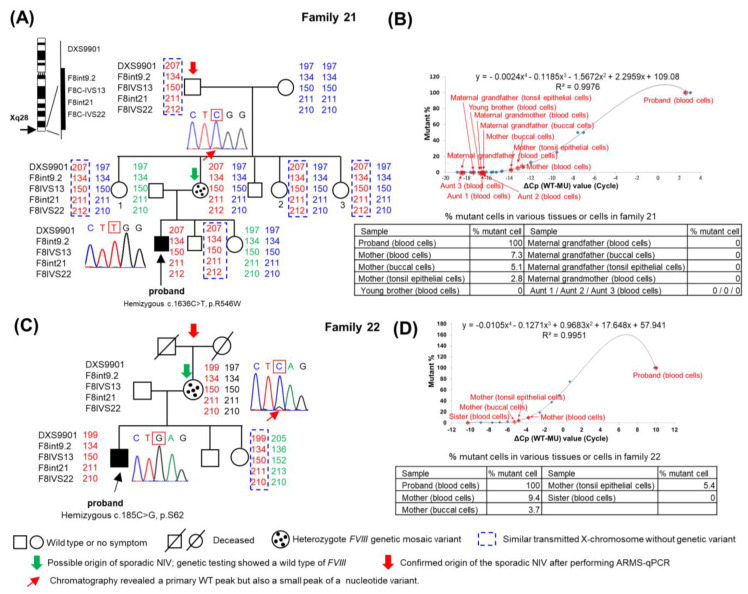
Linkage analysis using intra- and extragenic markers of *F8* gene, genetic testing, and amplification refractory mutation system–quantitative polymerase chain reaction (ARMS-qPCR) to confirm the origin of the sporadic mosaic noninversion variants (NIVs) in families 21 and 22. (**A**,**C**) Linkage analysis using *F8* gene markers in family 21, mild hemophilia A, and family 22, severe hemophilia A, respectively. The *F8* gene located on the X chromosome (Xq28) is shown on the left side of (**A**), along with the four intragenic markers and one extragenic marker (F8int9.2, F8IVS13, F8int21, F8IVS22, and DXS9901) selected for linkage analysis. (**B**,**D**) Determination of the percentage of mutant cells using ARMS-qPCR among various tissue cells obtained from family members to confirm the origin of the sporadic NIVs (~0% mutant cells) or the presence of somatic and/or germline mosaic variants (0–50% mutant cells) in families 21 and 22, respectively. ΔCp (WT − MU) represents the differences between the qPCR cycle crossing (Cp) of the wild-type (WT) and mutant (MU) alleles of different synthetic dilutions (X–axis). Blue points (◆) indicate various synthetic dilutions prepared through a two-fold serial dilution of MU DNA using WT DNA as described in the text. The standard curve shown in the upper part of each figure was generated by plotting the ΔCp value (X–axis) against known mutant percentages of various synthetic dilutions (Y−axis). Thereafter, the equations for X and Y were derived. When ΔCp (X) of the test sample after ARMS-qPCR was known (shown in red asterisks), Y (% mutant cells) could be calculated from each equation as shown in each table.

Linkage analysis and ARMS-qPCR were performed on the family members of the families 20–22, as described above. For example, as shown in Figure 2, the mother in family 21 (Figure 2A) and family 22 (Figure 2C) could be the possible origin of the sporadic NIV (shown in green arrow) according to linkage analysis and Sanger genetic testing. However, the resulting chromatographic analysis revealed not only a primary WT peak but also a small peak of a nucleotide variant. As shown in Figure 2B,D (table insets), ARMS-qPCR revealed 100% mutant cells in the blood cells of both probands, as expected; some mutant cells in various tissue cells of the mother of family 21 (Figure 2B) and of family 22 (Figure 2D) indicate that both mothers had mosaic variants. Therefore, neither mother was the origin of the sporadic NIV. Linkage analysis, genetic testing, and ARMS-qPCR further confirmed that the maternal grandfather of family 21 was the origin of the sporadic NIV (shown in red arrow) as he had a transmitted X chromosome similar to that of the mother and the proband while revealing a WT *F8*. Importantly, he showed 0% mutant cells in all tissue cells obtained. Interestingly, the maternal grandfather transmitted the same X chromosome to his three daughters, but none of them had mutant cells in the blood; the proband and another son inherited the same X chromosome from their mother, but the younger brother did not have the genetic variant. For family 22, the mother transmitted the same X chromosome to the proband and his sister, but the sister did not bear the genetic variant. Given that both maternal grandparents in family 22 were deceased, no further tests could be performed, and the confirmed origin remained undetermined.

Table 2 summarizes the family members initially designated as possible origins of sporadic NIVs via linkage analysis and genetic testing but showing mosaic variants via ARMS-qPCR for each family (families 20–22). Family members from earlier generations were further designated as the confirmed origins of the sporadic NIVs via linkage analysis, genetic testing, and the ARMS-qPCR assay. However, the maternal grandparents in family 22 were deceased; therefore, further testing was not possible.

### 2.3. Possible Timing of Sporadic NIV Occurrence during Embryonic Development

#### 2.3.1. Categorization of the Timing in Families 1–19

**Figure 3:**
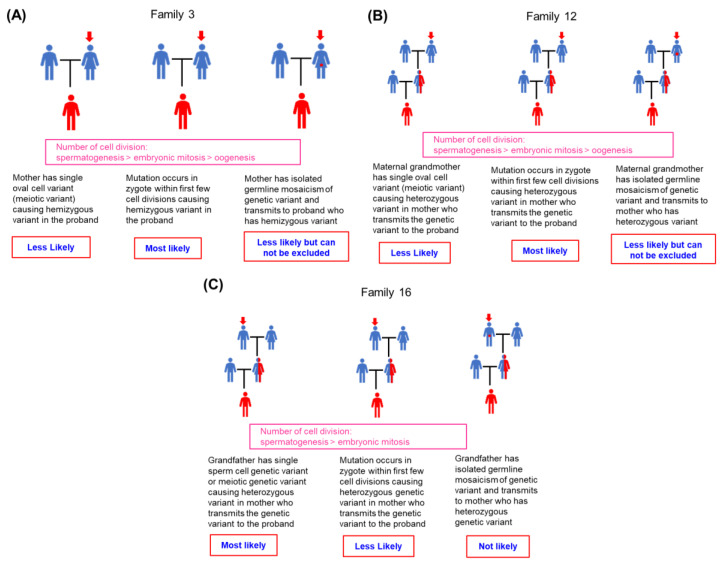
Possible timing of the occurrence of sporadic noninversion variants during embryogenesis. The most likely timing of the occurrence of sporadic noninversion variants during embryogenesis is described for each family and compared with other timing possibilities during embryogenesis based on the relative likelihood of occurrence according to the number of cell divisions and whether or not germ cells were available for testing as well as clinical data. The red arrow indicates the confirmed origin of the variant. (**A**) Family 3. (**B**) Family 12. (**C**) Family 16.

The possible timing of sporadic NIV occurrence during embryonic development in families 1–19 is summarized in Table 3. Examples of families 3, 12, and 16 are shown in Figure 3A–C, respectively. We considered that the sporadic NIV most likely occurred at the zygote stage within the first few cell divisions in the proband of family 3 and the mother of family 12, causing a hemizygous variant in the proband of family 3 and a heterozygous variant in the mother of family 12; the latter transmitted the genetic variant to the proband. The maternal grandfather of family 16 had a single sperm cell variant, resulting in a heterozygous status in the mother based on the relative likelihood of occurrence according to the number of cell divisions and clinical data. Other possibilities are presented, although they are less plausible or cannot be ruled out because no ovarian tissue was available for study in families 3 and 12. Regarding family 16, other possibilities are less likely, as the number of cell divisions is highest during spermatogenesis. Furthermore, the likelihood that the maternal grandfather has an isolated germline mosaicism is not likely, as his sperm cells showed 0% mutant cells.

#### 2.3.2. Categorization of Timing in Families 20–22

**Figure 4:**
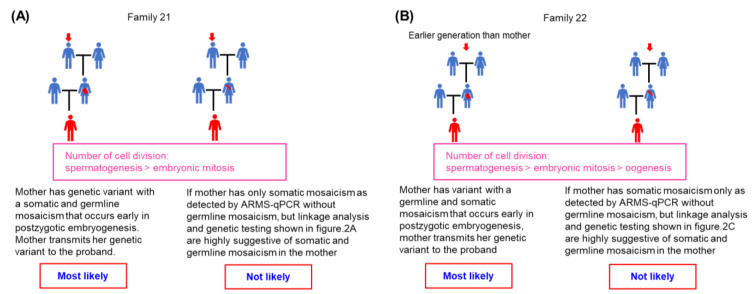
Possible timing of sporadic noninversion mosaic variant occurrence during embryogenesis. The most likely timing of the occurrence of sporadic noninversion mosaic variants during embryogenesis is described for each family and compared to other timing possibilities during embryogenesis based on the relative likelihood of occurrence according to the number of cell divisions and whether germ cells were available for testing as well as clinical data. The red arrow indicates the confirmed origin of variant. (**A**) Family 21. (**B**) Family 22.

The possible timing of sporadic NIV occurrence in families 20–22 is summarized in Table 3. Families 21 and 22 are shown as examples in Figure 4A,B, respectively. We observed that the genetic variant most likely occurred early in postzygotic development, causing both somatic and germline mosaic variants in the two mothers. The other possibility is unlikely because there would be mutant cells in these two mothers’ germline tissues, which would then transmit the variant to the proband; otherwise, two independent variant events (one in each mother and one in the respective proband) causing a similar variant in a family would be extremely rare.

## 3. Discussion

Sporadic hemophilia has been reported in 30–60% of PWH [8,16,17,18]. When hemophilia A sporadic NIV occurred in a proband; the proband’s mother was the first person to be evaluated and would be either determined to be free of the same HA-sporadic NIV (families 1–9, Table 1 and Table 3) or identified as an HA carrier (families 10–19, Table 3) or a family member bearing a mosaic genetic variant (families 20–22, Table 2 and Table 3); the maternal grandparents were the origin of sporadic NIVs in the latter two cases and were also shown to be free of the same HA-sporadic NIV (maternal grandmother, families 10–13 and 20, Table 1 and Table 2; maternal grandfather, families 14–19 and 21, Table 1 and Table 2). Every father of the proband would contribute a Y chromosome that did not bear *F8,* and every other spouse of maternal grandparents contributed a different X chromosome that did not bear sporadic NIV to carrier mothers; therefore, all maternal grandparents would be free of the same sporadic NIV confirmed by ARMS-qPCR and/or genetic testing except for two deceased maternal grandfathers (families 11 and 20) who were not the origin of HA-sporadic NIV. These results were compatible with the definition of a DNV, which stated that the origin must be free of genetic variants associated with DNVs (all parents of families 1–9 and all maternal grandparents of families 10–21) and verified that HA-sporadic NIV was caused by a DNV of the *F8*. In this study, we precisely demonstrated that DNVs occur in all families. Additionally, we identified the family members (probands of families 1–9; mothers of families 10–21; Table 3) who bear HA-sporadic NIVs, and their origin (mothers of families 1–9; maternal grandparents of families 10–21; Table 3) showed 0% mutant cells among blood and tissue cells derived from three germ layers of the embryogenic stage, except the one family (family 22) whose maternal grandparents were deceased, although no germinal tissues were available for studies. Those family members confirmed as the origins of HA-sporadic NIV or identified as carriers of HA were primarily mothers or maternal grandparents who did not recognize their status until one of their male descendants became the family’s first affected hemophilic boy; otherwise, they would not have known about the origin or carriership of HA. Each of the nine mothers who were not carriers of HA gave birth to only one hemophilic boy; thus, we were unable to determine whether any of them had isolated germline mosaicism as no germinal tissue was available for study. Both intra- and extragenic STR markers of the *F8* gene, which were used for linkage analysis, were more accurate than the restriction fragment length polymorphism analysis utilized in previous studies [10,15,16]. ARMS-qPCR, having a better variant detection sensitivity (<1%) [19,20,21], was used to confirm whether the family member was the real origin of sporadic NIV (Table 1 and Table 2).

While many technological advances allow detailed analysis of genetic makeup, such as next-generation sequencing (NGS), the detection and quantification of low-level mosaicism within the genetic composition is currently hindered by technical limitations of coverage depth and unique molecular identifiers (UMIs). Although promising NGS protocols have been developed, most methods are not sufficiently sensitive to detect low-level mosaic mutations within the vast background of wild-type DNA [22]. Currently, a variety of techniques are applied, all of which show some advantages and disadvantages, and ARMS-qPCR is no exception. Typical ARMS-qPCR may have false positive signals caused by primer mismatch at high background concentrations. Despite the base-pair mismatch, false positives are often caused by primer elongation and define the limit of sensitivity for each assay [23]. ARMS-qPCR using conventional primers and polymerases has been reported to achieve a detection limit of 1 variant in 1000 wild-type copies [24]. The modified protocol has been shown to have a sensitivity of detecting 1 mutant DNA copy in 200,000 wild-type DNA copies [25]. The main advantages of the ARMS-qPCR method we have used are (1) high sensitivity (at least <0.1% limitation); (2) flexible input DNA amount, thereby eliminating the requirement of further processing; (3) flexibility to customize for each family-specific mutant; and (4) a simple and cost-effective protocol. Nevertheless, ARMS-qPCR remains limited. We considered that the origin of sporadic NIV is not the occurrence of sporadic NIV, and the two events should be distinct from each other, as shown in Table 3, so that sporadic NIV can be correctly investigated [14]. Sporadic NIV can occur either before or after conception [13,14]; therefore, it is impossible to examine a single embryo and determine the exact timing of the occurrence of the sporadic NIV. Therefore, we determined the most likely timing at which the sporadic NIV occurred, as described in Table 3 and Figure 3 and Figure 4.

Sporadic variants of the *F8* gene appear to be one-time occurrences, as shown in Figure 1A,C,E. The mother (family 3), maternal grandmother (family 12), and maternal grandfather (family 16) transmitted the same X chromosome to the proband and their other offspring (sister in family 3; one aunt and one uncle in family 12; and aunt in family 16), but the genetic variant was absent in all other offspring. DNV, or HA-sporadic NIV, is an incidental episode that causes a one-time event; however, transmission of one or more *F8* genetic variants in family members with familial HA is inevitable. These findings are consistent with the concept presented in Figure 3A–C. Figure 2A (family 21) and 2B (family 22) showed an interesting finding that the same X chromosome got transmitted from each mother to two children, where one had a genetic variant (proband), but the other did not. These findings suggest that these two mothers had germline mosaic variants. Previous studies have reported that a mother of a proband who did not have the variant gave birth to more than one child carrying the same variant; a germline mosaicism was suggested [8,26]. Given the diverse approaches used to detect somatic and/or germline mosaicism, it was found in 13.6% (3 of 22) of the families. Similarly, somatic mosaicism has been found to be a fairly common occurrence in HA (13%) [27]. The peculiar pattern of chromatography is highly suggestive of the fact that a mosaic variant could easily be overlooked, as Sanger sequencing has low sensitivity [28,29]. Additionally, genetic mosaicism has been reported in other hereditary diseases [30,31,32].

As we mentioned above, sporadic hemophilia A is seen fairly commonly in clinical hemophilia care practice. Why only one genetically normal mother gave birth to a hemophilic boy and why only one mother became a carrier of hemophilia among her sisters is an unexplainable question we have encountered for many years. Now, we are pleased to be able to give a scientific and reasonable answer. Similar studies can be extended to other monogenic disorders to understand the genetic pathogenesis if linkage analyses and the measurement of percent mutant cells can be performed.

One limitation of this study is that when discriminatory primers for nonspecific variant regions and/or homozygous recombination (such as INV1 and INV22 variants in HA) cannot be created, the ARMS-qPCR technique cannot be used. Other limitations are that germinal tissues are almost unavailable for study, especially from female family members to show germline genetic variants or isolated germline mosaicism, and there is difficulty in recruiting families for study. A relatively small number of eligible families were investigated, and more sporadic hemophilia families are needed for studies in the future to solve the question of whether there is a possibility of germline genetic variants or genetic mosaicism in mothers or maternal grandparents.

## 4. Materials and Methods

### 4.1. Patient and Family Groups and Study Design

**Figure 5:**
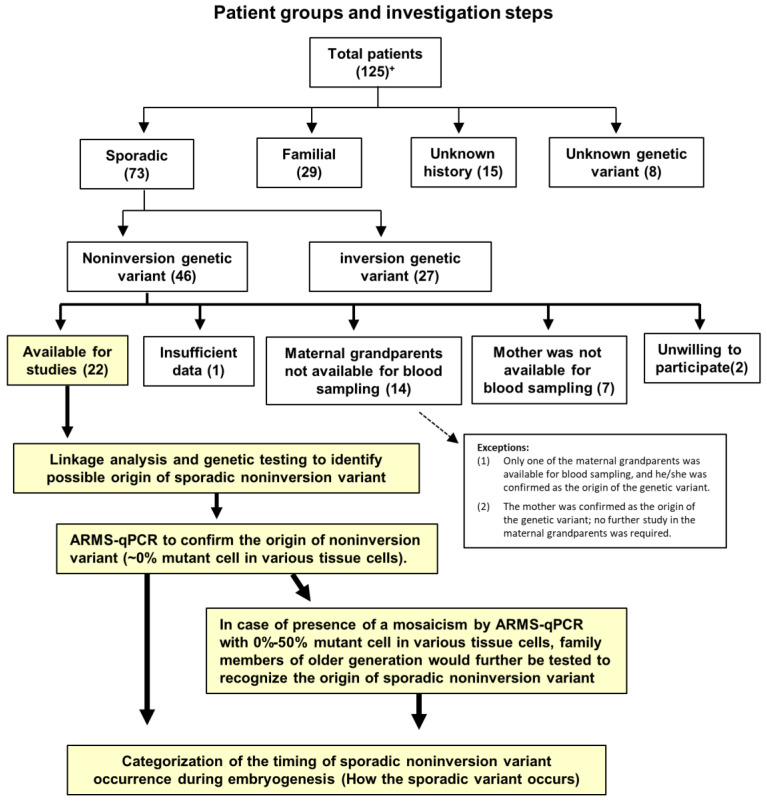
Patient groups and investigation steps. Sporadic patients were recruited for studies from all registered patients with hemophilia A. Many patients were excluded, as shown. Linkage analysis and genetic testing to identify the possible origin of sporadic noninversion variants (NIVs), amplification refractory mutation system–quantitative polymerase chain reaction (ARMS-qPCR) were conducted to confirm the origin of sporadic NIVs or mosaic variants, and categorization of the timing of occurrence of sporadic NIVs during embryogenesis are all described in the text. + Number of patients.

This study was performed in accordance with the Declaration of Helsinki and approved by the Institutional Review Board of Changhua Christian Hospital (approval no. 201209). All patients and their family members provided informed consent. As shown in Figure 5, to avoid selection bias, sporadic patients were recruited from the year of 2015 among the 125 registered families with HA. Most of them had three generations (patients to maternal grandparents). Several patients were excluded for certain reasons, as shown in Figure 5. Finally, 22 patients and their family members were eligible for studies on the actual origin and timing of hemophilia A sporadic NIV.

### 4.2. Collection of Blood and Tissue Cells from Patients and Family Members

Palatine tonsil epithelial cells, blood cells, and buccal cells are derivatives of the endoderm, mesoderm, and ectoderm, respectively [33,34]. The DNA from blood and tissue cells, or sperm cells if available, was isolated for *F8* genetic analysis and detection of the percent mutant cells by ARMS-qPCR as described below. These three embryonic germ layers will form organs and tissues of the embryo, and the other branch, primordial germ cells, will form gonads.

### 4.3. DNA Extraction from Blood and Tissue Cells

Genomic DNA was isolated from peripheral blood cells, buccal cells, tonsil epithelial cells, and sperm cells according to the supplier’s protocol of the QIAamp^®^ DNA Investigator kit (QIAGEN, Hilden, Germany). Briefly, samples were lysed using pulse–vortexing and the lysates were subsequently transferred to the QIAamp MinElute column to bind with DNA. To ensure efficient binding, ethanol was added. QIAamp MinElute column was washed twice before DNA was eluted from the column, and the DNA was ready for use. The quality and purity of DNA were evaluated based on the absorbance values and ratios at 260 nm and 280 nm using a Nanodrop 2000 spectrophotometer (Thermo Fisher Scientific, Waltham, MA, USA) [35].

### 4.4. Diagnosis of Hemophilia A

A genetic analysis was performed to screen for the *F8* variant. An inverse polymerase chain reaction (I-PCR) and multiplex PCR were performed to detect the common IVS22 and IVS1 of *F8* gene [35,36]. Sanger sequencing was performed to detect variants on the exons and exon-intron junctions of *F8* gene. The nomenclature for the description of sequence variants follows the guidelines of the Human Genome Variation Society (https://www.hgvs.org/; accessed on 10 August 2022). Multiplex ligation-dependent probe amplification (MLPA) was performed using the SALSA MLPA Probemix P178 *F8* to detect the underlying deletions that may escape detection by PCR-based analysis due to masking of the wild-type allele. Analysis of exon dosages by MLPA was described in the previous study [36].

### 4.5. Linkage Analysis and Genetic Testing for the Identification of the Possible Origin of Sporadic NIVs

*F8* is X chromosome-linked; therefore, the origin of sporadic NIVs could be identified through linkage analysis using intra- and extragenic HA gene markers (short tandem repeats, STRs). Some intragenic markers of HA are F8int9.2, F8IVS13, F8int21, and F8IVS22, whereas DXS9901 is an extragenic marker [36,37,38]. Capillary electrophoresis using a GenomeLabTM GeXP Genetic Analysis System (Beckman Coulter, Brea, CA, USA) under routine running conditions was performed. Subsequently, data were analyzed using the Beckman Coulter fragment analysis package (software version 10.2.3; Algorithm version 3.2.42). The same size of each fragment for the five STR markers would be considered the same transmitted X chromosome. A family member who was a candidate for the origin of a sporadic NIV as determined by linkage analysis and showed a wild-type *F8* as determined by genetic testing would be identified as the possible origin of the sporadic NIV.

### 4.6. Amplification Refractory Mutation System–Quantitative Polymerase Chain Reaction

**Figure 6:**
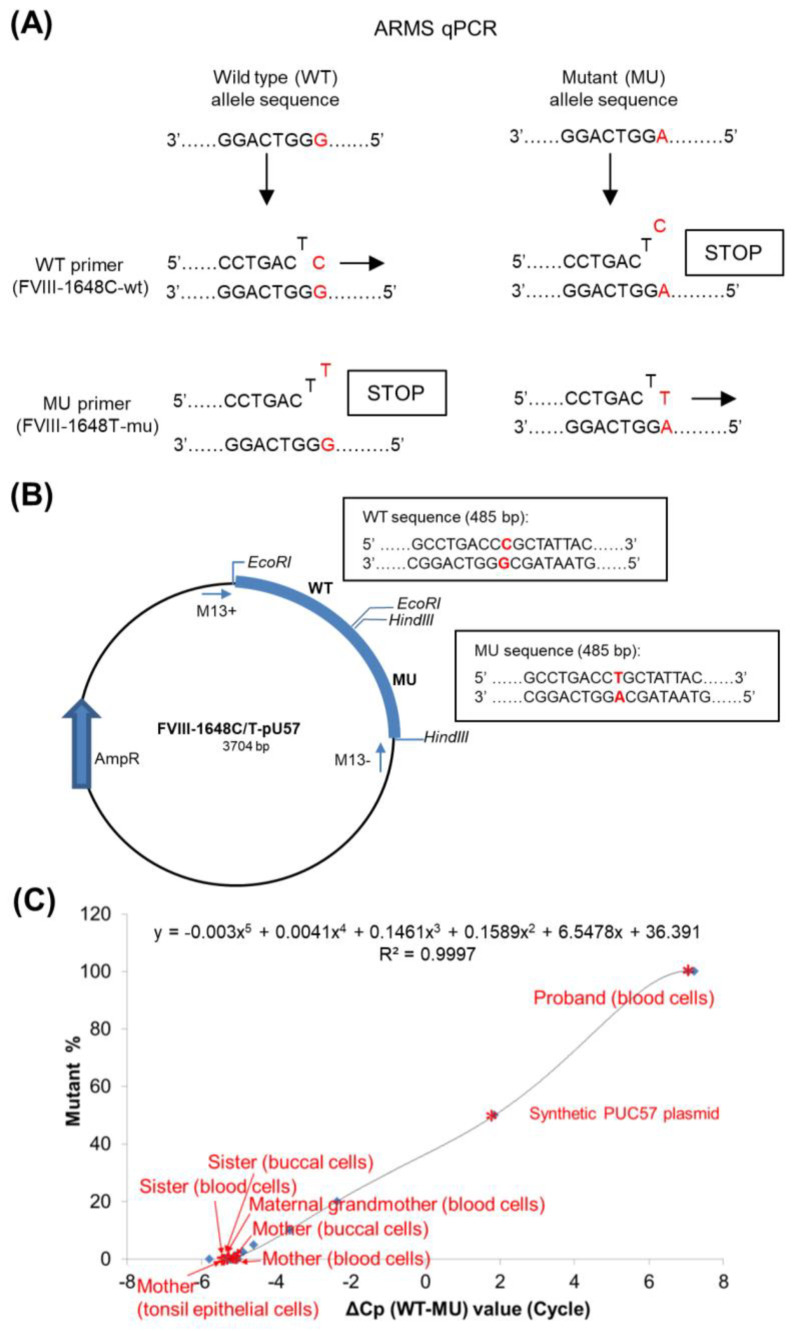
Schematic representation of amplification refractory mutation system–quantitative polymerase chain reaction (ARMS-qPCR) of the family with the *F8* c.1648C>T variant. (**A**) ARMS-qPCR in a case with *F8* c.1648C>T variant to identify the mutant (MU) and wild-type (WT) alleles using WT- and MU-specific primers. (**B**) The synthetic plasmid (*F8*-1648C/T-pUC57) has a pUC57 plasmid backbone that contains the ampicillin AmpR gene, a mutant (MU) *F8* sequence, and a wild-type (WT) *F8* sequence. The inserted sequence was cloned into pUC57 using an *EcoRV* restriction site; the insertion was confirmed via bidirectional sequencing primers (M13+: AGGGTTTTCCCAGTCACG/M13−: GAGCGGATAACAATTTCACAC). Specific *EcoRI* and *HindIII* restriction enzyme cleavage sites flanked the WT and MU sequences, respectively. (**C**) A standard curve for ARMS-qPCR, which was generated by plotting X (X− axis) against Y (Y− axis). An equation for X and Y was then derived as shown in the upper part of the figure. X was unknown, to be determined by measurement of the difference between the PCR cycle crossing point (Cp) of the WT and MU alleles, i.e., (ΔCp, WT − MU) value of different synthetic dilutions shown in blue points. Y was known, the percent mutant cells of the synthetic dilution prepared by 2-fold serial dilution of MU DNA by WT DNA and calibrated by synthetic plasmid. To measure the percentage of mutant cells in a DNA test sample, determine its ΔCp (X−value) first shown using red asterisks, then percent mutant cells (Y− value) can be derived from the equation.

Amplification refractory mutation system–quantitative polymerase chain reaction (ARMS-qPCR) is a customized PCR method that differentially amplifies mutant (MU) and wild-type (WT) alleles with enhanced sensitivity for detecting low levels of mutant cells. For this reason, we employed the ARMS-qPCR method to explore the origin of family variants. Two sequence-specific forward primers modified with a mismatch at the penultimate nucleotide position of the variant site were designed. For example, the following primers were used in the analysis of family 3 containing the c.1648C>T (p.Arg550Cys) variant of *F8* as shown in Figure 6A: (F8-1648T-mu: 5′-CAGATCCTCGGTGCCTGACTT-3′ for the MU allele and F8-1648C-wt: 5′-CAGATCCTCGGTGCCTGACTC-3′ for the WT allele). The two forward primers were paired with the same reverse primer F8-1648-R: 5′-CAGCACTTGGAAAGGCAAGAAC-3′ to produce an equivalent 151 bp product. The primers used for each family are shown in Table S1.

After confirmation by familial sequencing analysis, the DNA of the male proband and the DNA of the male healthy individual were selected as the sources of 100% mutant and full WT samples, respectively. Then, the two samples were amplified against the target region of the variant [36], and a series of dilutions were performed to prepare the samples required for subsequent standard curves. In addition, a synthetic PUC57 plasmid containing a WT *F8* sequence and a MU *F8* sequence were included as a standard representing a 50% variant, as shown in Figure 6B; this standard was diluted appropriately for curve calibration. Individual pUC57 plasmids were synthesized for each different genetic variant.

All DNA samples, including serially diluted samples, family member samples, and the synthetic plasmid, were scheduled in the same ARMS-qPCR experiment, which was performed in triplicate on a Light Cycler^®^ 480 Real-Time PCR System (Roche, Rotkreuz, Switzerland). Each 20-μL reaction mixture consisted of 30 ng of template DNA, 0.3 μmol/L of each primer, and 1× Smart Quant Green Master Mix (Protech Technology Enterprise Co., Taipei, Taiwan). PCR conditions were as follows: 95 °C for 10 min, followed by 45 cycles at 95 °C for 10 s and 60 °C for 30 s. [14]. The standard curve of the family is presented in Figure 6C.

### 4.7. Categorization of the Possible Timing of Sporadic NIVs during Embryogenesis

Sporadic NIVs occurred at one of several time periods during embryogenesis, which were categorized as previously described [13,14]: variants occurring during the single germ cell stage, those occurring during the first few cell divisions of the zygote stage, and those occurring early in postzygotic development. Variants that occur during the first two time periods lead to a complete variant in the offspring, whereas the third variant timing leads to a mosaic variant in the offspring. The variant rate is correlated with the number of cell divisions [39,40]; thus, spermatogenesis has a higher rate of spontaneous variation than embryonic mitosis, which has a higher rate than oogenesis. Other mechanisms that produce a high variant rate may include DNA damage repair and exposure to mutagens.

The timing of the initial NIV was categorized after considering two facts: first, who among the family members was the origin of the sporadic NIV, and second, whether the sporadic NIV was a complete or mosaic variant.

## 5. Conclusions

We performed the origin and timing study for HA-sporadic NIVs. We found that DNVs in the *F8* gene cause sporadic hemophilia A. We demonstrated that somatic and germline mosaicism occurred in 13.6% of the families under study (3 out of 22). These findings contribute to a better knowledge of the genetic pathogenesis of HA, which is crucial for improving genetic counseling for this disease.

## Figures and Tables

**Table 1:** Characteristics of the proband and percent mutant cells as determined by amplification refractory mutation system–quantitative polymerase chain reaction (ARMS-qPCR) in tissue cells obtained from family members designated as a possible and confirmed origin of hemophilia A sporadic noninversion variant (NIV).

Family No. and Age of Proband (Year)	Family File No.	FVIII Level (IU/dL)	Exon	Nucleotide Change	Amino Acid Substitution	Family Members Designated as the Possible Origin of Sporadic NIVs	Percentage of Mutant Cells by ARMS-qPCR in Tissue Cells Obtained from Family Members Designated as the Possible Origin of Sporadic NIVs			Family Members Designated as the Confirmed Origin of Sporadic NIVs	Percentage of Mutant Cells by ARMS-qPCR in Tissue Cells Obtained from Family Members Designated as Confirmed Origin of Sporadic NIVs		
							Blood Cells	Buccal Cells	Tonsil Epithelial Cells		Blood Cells	Buccal Cells	Tonsil Epithelial Cells
1 (16)	74	<1	19	c.6046C>G	p.R2016G	M	0	0	0	M	0	0	0
2 (57)	31	<1	7	c.822G>T	p.W274C	M	0	0	0	M	0	0	0
3 (9)	189	<1	11	c.1648C>T	p.R550C	M	0	0	0	M	0	0	0
4 (15)	176	3.7	14	c.5122C>T	p.R1708C	M	0	0	0	M	0	0	0
5 (6)	208	<1	20	c.6131T>C	p.L2044P	M	0	0	0	M	0	0	0
6 (11)	209	1.0	14	c.4379delA	p.N1460Ifs*5	M	0	0	0	M	0	0	0
7 (48)	NTUH	<1	9	c.1412T>A	p.L471*	M	0	0	0	M	0	0	0
8 (26)	147	<1	4	c.403G>A	p.D135N	M	0	0	0	M	0	0	0
9 (4)	180	<1	14	c.2945dupA	p.N982Kfs*9	M	0	0	0	M	0	0	0
10 (23)	referred-1	<1	14	c.2945dupA	p.N982Kfs*9	MGM	0	0	0	MGM	0	0	0
11 (22)	155	<1	15	c.5343T>A	p.Y1781*	MGM	0	0	0	MGM	0	0	0
12 (19)	78	<1	14	c.3637delA	p.I1213Ffs*5	MGM	0	0	0	MGM	0	0	0
13 (7)	131	1.2	IVS10	c.1538-1G>A	-	MGM	0	0	0	MGM	0	0	0
14 (2)	207	<1	12	c.1848dupT	p.P617Sfs*7	MGF	0	0	0	MGF	0	0	0
15 (0.1)	0	<1	IVS14	c.5219+1G>A	-	MGF	0	0	0	MGF	0	0	0
16 (5)	referred-2	<1	12	c.1813T>C	p.Y605H	MGF+	0	0	0	MGF+	0	0	0
17 (5.4)	187	<1	14	c.2322delA	p.Q774Hfs*12	MGF	0	0	0	MGF	0	0	0
18 (2)	NTUH-2	<1	14	c.3637dupA	p.I1213Nfs*28	MGF	0	0	0	MGF	0	0	0
19 (7)	211	<1	23	c.6548_6554delTGGAGTT	p.M2183Rfs*9	MGF	0	0	0	MGF	0	0	0

M, mother; MGM, maternal grandmother; MGF, maternal grandfather. + Sperm exhibited 0% mutant cells.

**Table 2:** Characteristics of the proband and percentage of mutant cells as determined by amplification refractory mutation system–quantitative polymerase chain reaction (ARMS-qPCR) in tissue cells obtained from family members designated as a possible and confirmed origin of hemophilia A sporadic mosaic noninversion variant (NIV).

Family No. and Age of Proband (Year)	Family File No.	FVIII Level (IU/dL)	Exon	Nucleotide Change	Amino acid Substitution	Family Members Designated as the Possible Origin of Sporadic NIVs	Percentage of Mutant Cells by ARMS-qPCR among Tissue Cells Obtained from Family Members Designated as the Possible Origin of Sporadic Mosaic NIVs			Family Members Designated as the Confirmed Origin of Sporadic NIVs	Percentage of Mutant Cells by ARMS-qPCR among Tissue Cells Obtained from Family Members Designated as Confirmed Origin of Sporadic Mosaic NIVs		
							Blood Cells	Buccal Cells	Tonsil Epithelial Cells		Blood Cells	Buccal Cells	Tonsil Epithelial Cells
20 (10)	153	<1	10	c.1525A > T	p.R509*	M	18.1	23.8	24.1	MGM	0	0	0
21 (38)	130	25.1	11	c.1636C > T	p.R546W	M	7.3	5.1	2.8	MGF	0	0	0
22 (47)	105	<1	2	c.185 C > G	p.S62*	M	9.4	3.7	5.4	EGT M	NA	NA	NA

M, mother; MGM, maternal grandmother; MGF, maternal grandfather; EGT, earlier generation than; NA: not available.

**Table 3:** Origin, occurrence, and timing of the noninversion variant (NIV) of the *F8* gene during embryonic development in sporadic hemophilia A.

Family No.	Family Members Designated as the Confirmed Origin of Sporadic NIV	Family Members in Whom Sporadic NIVs Occurred	Timing of the Sporadic NIV Occurrence during Embryonic Development
1	M	Proband	Genetic variant most likely occurs in the zygote within the first few cell divisions, thereby causing a hemizygous variant in the proband. ^†^
2	M		
3	M		
4	M		
5	M		
6	M		
7	M		
8	M		
9	M		
10	MGM	Mother	Genetic variant most likely occurs in the zygote within the first few cell divisions, thereby causing a heterozygous variant in the mother who transmits the genetic variant to the proband. ^‡^
11	MGM		
12	MGM		
13	MGM		
14	MGF	Mother	Maternal grandfather most likely has a genetic variant in a single sperm cell, thereby causing a heterozygous variant in the mother who transmits the genetic variant to the proband. ^§^
15	MGF		
16	MGF		
17	MGF		
18	MGF		
19	MGF		
20	MGM	Mother+	Genetic mosaic variant most likely occur during the early stages of embryogenesis, thereby causing a somatic and germline mosaic genetic variant in the mother who transmits the genetic variant to the proband. ^¶^
21	MGF		
22	EGT M		

M, mother; MGM, maternal grandmother; MGF, maternal grandfather; EGT, earlier generation than. + Family member who bears a mosaic genetic variant. ^†^ Examples of other possibilities are shown and evaluated in Figure 3A. ^‡^ Examples of other possibilities are shown and evaluated in Figure 3B. ^§^ The number of cell divisions is highest during spermatogenesis; examples of other possibilities are shown and evaluated in Figure 3C. ^¶^ Examples of other possibilities are shown and evaluated in Figure 4.

## Data Availability

The data reported in this study are available upon request from the corresponding author. These data are not publicly available due to ethical restrictions.

## Supplementary Materials

The following supporting information can be downloaded at https://www.mdpi.com/article/10.3390/ijms25031763/s1.
