# Supplementary material for: De Novo Noninversion Variants Implicated in Sporadic Hemophilia A: A Variant Origin and Timing Study
Source: Int J Mol Sci. 2024 Feb 1;25(3):1763. doi: 10.3390/ijms25031763 (PMC10855912; doi:10.3390/ijms25031763)
Supplement: Supplementary file 1 [file ijms-25-01763-s001.zip › ijms-2746983-Supplementary.pdf]

International Journal of *Molecular Sciences*

MDPI

Supplemental Table S1. Primer pairs used for ARMS-qPCR

Family	Mutant	Forward primer	Sequence 5′→ 3′	Tm	Reverse primer	Sequence 5′→ 3′	Tm	size (bp)	Genomic position (hg38)
1	F8 (c.6046C>G)	F8-6046C-wt	CCATCCAAAGCTGGAATTTG**A**C	55.1	F8-6046-R	GAACCTCTGCCCACATTGCTAC	55.2	117	>chrX:154902025-154902141
		F8-6046G-mu	CCATCCAAAGCTGGAATTTG**A**G	55.3					
2	F8 (c.822G>T)	F8-822G-wt	GCCACAGGAAATCAGTCTAT**C**GG	56.5	F8-822-R	CCAAGGTCCATCAAGAGTGTTTG	55.1	169	>chrX:154969372-154969540
		F8-822T-mu	GCCACAGGAAATCAGTCTAT**C**GT	54					
3	F8 (c.1648C>T)	F8-1648C-wt	CAGATCCTCGGTGCCTGAC**T**C	56	F8-1648-R	CAGCACTTGGAAAGGCAAGAAC	55.5	151	>chrX:154956931-154957081
		F8-1648T-mu	CAGATCCTCGGTGCCTGAC**T**T	55.6					
4 *	F8 (c.5122C>T)	F8-5122-F	GGCAAAGCAAGGTAGGACTGAA	55.1	F8-5122C-wt	GTCGTGTTTTCTTTTGAAAGCTG**T**G	56.2	212	>chrX:154928644-154928855
					F8-5122T-mu	GTCGTGTTTTCTTTTGAAAGCTG**T**A	54		
5	F8 (c.6131T>C)	F8-6131T-wt	ATTTCAGAGTGTCAGACTCCC**G**T	53.5	F8-6131-R	ATAATCAGCCCAGGTTCTTGGAG	55.4	121	>chrX:154901329-154901449
		F8-6131C-mu	ATTTCAGAGTGTCAGACTCCC**G**C	56.2					
6	F8 (c.4379delA)	F8-4379-wt	TTTCTTACAAGGAGCCAAAAAA**G**AT	54.1	F8-4379-R	AGATGTTTTGGGCAAGTCTGGTT	55.7	168	>chrX:154929266-154929434
		F8-4379delA-mu	TTTCTTACAAGGAGCCAAAAAA**G**TA	53.1				167	
7	F8 (c.1412T>A)	F8-1412T-wt	GAATCAGGAATCTTGGGACCT**C**T	54.3	F8-1412-R	CCATTGGAGACAAGGCTGAATTA	54.9	151	>chrX:154965873-154966023
		F8-1412A-mu	GAATCAGGAATCTTGGGACCT**C**A	55.3					
8	F8 (c.403G>A)	F8-403G-wt	CCTGCTATAGGAGCTGAATATGA**A**G	52.9	F8-403-R	TAGGTAAGGCACAGTGGGTCAG	53.4	146	>chrX:154993013-154993158
		F8-403A-mu	CCTGCTATAGGAGCTGAATATGA**A**A	53.2					
9	F8 (c.2945dupA)	F8-2945A-wt	CAAGAAAGTTCATGGGGAAAAA**G**T	54.7	F8-2945-R	AGTCAACAAAGCAGGTCCATGAG	54.9	90	>chrX:154930778-154930867
		F8-2945dupA-mu	CAAGAAAGTTCATGGGGAAAAA**G**A	55.5				91	
10	F8 (c.2945dupA)	F8-2945A-wt	CAAGAAAGTTCATGGGGAAAAA**G**T	54.7	F8-2945-R	AGTCAACAAAGCAGGTCCATGAG	54.9	90	>chrX:154930778-154930867
		F8-2945dupA-mu	CAAGAAAGTTCATGGGGAAAAA**G**A	55.5				91	

11	F8 (c.5343T>A)	F8-5343T-wt	GGACTCCTGGGGCCAT**G**T	54.1	F8-5343-R	CCACTGTCCTTAACTCACCATGATA	54	66	>chrX:154906402-154906467
		F8-5343A-mu	GGACTCCTGGGGCCAT**G**A	55.2					
12	F8 (c.3637delA)	F8-3637-wt	AAATAATACACACAATCAAGAAAAAAA**G**A	53	F8-3367-R	GAGGCAAAACTACATTCTCTTGG	54.4	87	>chrX:154930095-154930181
		F8-3637delA-mu	AAATAATACACACAATCAAGAAAAAAA**G**T	52.3				86	
13	F8 (c.1538-1G>A)	F8-1538-1G-wt	TATGGTTTTGCTTGTGGGT**G**G	54.8	F8-1538-R	TGAGGAGAGGGCCAATGAGT	53.4	198	>chrX:154956995-154957192
		F8-1538-1A-mu	TATGGTTTTGCTTGTGGGT**G**A	53					
14	F8 (c.1848dupT)	F8-1848-wt	TACAACGCTTTCTCCCCAA**C**C	55.8	F8-1848-R	ATGTTGGAGGCTTGGAACTCTG	54.8	67	>chrX:154953900-154953966
		F8-1848dupT-mu	TACAACGCTTTCTCCCCAA**C**T	53.1					
15*	F8 (c.5219+1G>A)	F8-5219+1F	AGTGGAGAGGCTCTGGGATTATG	55.4	F8-5219+1G-wt	AAGGAATAACCAATGCATTCAT**G**C	57.3	82	>chrX:154928546-154928627
					F8-5219+1A-mu	AAGGAATAACCAATGCATTCAT**G**T	54.9		
16	F8 (c.1813T>C)	F8-1813T-wt	ATTTGATGAGAACCGAAGCTG**A**T	53.7	F8-1813-R	TTCTTTATTCACCACCCACTGGA	55	157	>chrX:154953848-154954004
		F8-1813C-mu	ATTTGATGAGAACCGAAGCTG**A**C	54.1					
17	F8 (c.2322delA)	F8-2322-wt	CCTAGCACTAGGCAAAAGC**G**A	54.1	F8-2322-R	TGTCGCAAGAGCATCAACAAAT	54.8	143	>chrX:154931346-154931488
		F8-2322delA-mu	CCTAGCACTAGGCAAAAGC**G**T	53.3				142	
18	F8 (c.3637dupA)	F8-3637-wt	AAATAATACACACAATCAAGAAAAAAAA**G**T	53.5	F8-3367-R	GCCCCGTCATATGAACCTTCTAC	55.6	177	>chrX:154930005-154930181
		F8-3637dupA-mu	AAATAATACACACAATCAAGAAAAAAAA**G**A	54.1					
19	F8 (c.6548_6554delTGGAGTT)	F8-6548-wt	CGCAGCACTCTTCGC**G**TG	56.3	F8-6548del7-R	CTACCCATGGTTGAGGGAAGAAG	55.7	90	>chrX:154863036-154863125
		F8-6548del7-mu	CGCAGCACTCTTCGC**G**GA	58.2					
20	F8 (c.1525A>T)	F8-1525A-wt	GATGTCCGTCCTTTGTATTCAAG**A**A	55.4	F8-1525-R	ACAGCTGGAGAAAGGACCAACA	55.2	139	>chrX:154960973-154961111
		F8-1525T-mu	GATGTCCGTCCTTTGTATTCAAG**A**T	54.5					
21	F8 (c.1636C>T)	F8-1636C-wt	GGGCCAACTAAATCAGATCC**A**C	54.1	F8-1636-R	GATGAGGAGAGGGCCAATGAGT	55.9	102	>chrX:154956993-154957094

		F8-1636T-mu	GGGCCAACTAAATCAGATCC**A**T	53.7					
22	F8 (c.185C>G)	F8-185C-wt	AAATCTTTTCCATTCAACACC**C**C	55.1	F8-185-R	CCTTGGCTTAGCGATGTTGAAA	56	90	>chrX:154999492-154999581
		F8-185G-mu	AAATCTTTTCCATTCAACACC**C**G	56					

Bold and underlined: mismatches designed near the familial variants were based on the principle of destabilization strength for ARMS-qPCR. Most of the mismatches lined up at the penultimate nucleotide position of the wild-type (wt) and mutant (mu) primers. To enhance the discrimination between the two primers, mismatches for families 2, 6, and 19 were arranged at the third-from-last nucleotide position.

Asterisk: the designed primers were based on the corresponding antisense sequences, which allowed for proper mismatches and similar Tm values between the wt and mu primers and avoided the interference of the development of secondary structures.
